# Assessing the suitability of summary data for two-sample Mendelian randomization analyses using MR-Egger regression: the role of the $I^{2}$ statistic

**DOI:** 10.1093/ije/dyw220

**Published:** 2016-09-11

**Authors:** Jack Bowden, Fabiola Del Greco M, Cosetta Minelli, George Davey Smith, Nuala A Sheehan, John R Thompson

**Affiliations:** 1 MRC Integrative Epidemiology Unit, University of Bristol, Bristol, UK; 2 MRC Biostatistics Unit, Cambridge, UK; 3 Center for Biomedicine, EURAC research, Bolzano, Italy; 4 Respiratory Epidemiology, Occupational Medicine and Public Health, Imperial College London, London, UK; 5 Department of Health Sciences, University of Leicester, Leicester, UK

**Keywords:** Mendelian randomization, MR-Egger regression, measurement error, I^2^ statistic, simulation extrapolation

## Abstract

**Background**: MR-Egger regression has recently been proposed as a method for Mendelian randomization (MR) analyses incorporating summary data estimates of causal effect from multiple individual variants, which is robust to invalid instruments. It can be used to test for directional pleiotropy and provides an estimate of the causal effect adjusted for its presence. MR-Egger regression provides a useful additional sensitivity analysis to the standard inverse variance weighted (IVW) approach that assumes all variants are valid instruments. Both methods use weights that consider the single nucleotide polymorphism (SNP)-exposure associations to be known, rather than estimated. We call this the `NO Measurement Error' (NOME) assumption. Causal effect estimates from the IVW approach exhibit weak instrument bias whenever the genetic variants utilized violate the NOME assumption, which can be reliably measured using the F-statistic. The effect of NOME violation on MR-Egger regression has yet to be studied.

**Methods:** An adaptation of the $I^{2}$ statistic from the field of meta-analysis is proposed to quantify the strength of NOME violation for MR-Egger. It lies between 0 and 1, and indicates the expected relative bias (or dilution) of the MR-Egger causal estimate in the two-sample MR context. We call it $I_{GX}^{2}$. The method of simulation extrapolation is also explored to counteract the dilution. Their joint utility is evaluated using simulated data and applied to a real MR example.

**Results:** In simulated two-sample MR analyses we show that, when a causal effect exists, the MR-Egger estimate of causal effect is biased towards the null when NOME is violated, and the stronger the violation (as indicated by lower values of $I_{GX}^{2}$), the stronger the dilution. When additionally all genetic variants are valid instruments, the type I error rate of the MR-Egger test for pleiotropy is inflated and the causal effect underestimated. Simulation extrapolation is shown to substantially mitigate these adverse effects. We demonstrate our proposed approach for a two-sample summary data MR analysis to estimate the causal effect of low-density lipoprotein on heart disease risk. A high value of $I_{GX}^{2}$ close to 1 indicates that dilution does not materially affect the standard MR-Egger analyses for these data.

**Conclusions**: Care must be taken to assess the NOME assumption via the $I_{GX}^{2}$ statistic before implementing standard MR-Egger regression in the two-sample summary data context. If $I_{GX}^{2}$ is sufficiently low (less than 90%), inferences from the method should be interpreted with caution and adjustment methods considered.

## Introduction

Mendelian randomization (MR)^1^ has become an established method for probing questions of causality in observational epidemiology. By making use of genetic variants satisfying the instrumental variable (IV) assumptions, it is possible to test whether an exposure causally influences a health outcome by circumventing the problem of confounding that compromises standard associational methods. The explosion in publicly available summary data estimates of genetic association from large international genome-wide association (GWA) consortia^2^^,^^3^ has made MR ever more popular for two reasons. First, summary data estimates of causal effect from multiple genetic variants can be simply and transparently combined to yield results that closely mirror what would be obtained with individual participant data.^4^^,^^5^ Second, a dramatic rise in the number of variants available for the analysis has led to an increased power for testing causal hypotheses.^6^ In this paper we focus on the most common form of summary data MR study, whereby genetic associations with the exposure and outcome are gleaned from independent samples to furnish a `two-sample' analysis.^4^

The standard method for MR with summary data [referred to as the standard inverse variance weighted (IVW) approach^5^^,^^7^] makes the fundamental assumption that each included variant is a valid IV. That is, it is (i) associated with the exposure, (ii) not associated with any confounders of the exposure and outcome, and (iii) is only associated with the outcome through the exposure (see Figure 6 available as Supplementary data at IJE online). If (i–(iii) hold, then an inverse variance weighted average of the individual causal effect estimates (e.g. as in a meta-analysis) is both efficient and unbiased. Unfortunately, assumptions (ii)–(iii) are unlikely to hold in an MR study, particularly in the summary data setting when large numbers of variants are harvested from GWA studies and included in the analysis. For example, as part of a repertoire of MR analyses, Holmes *et al*.^8^ conduct an MR-analysis of high density lipoprotein cholesterol on heart disease risk by liberally including many genetic variants that were also associated with other lipid fractions (e.g. triglycerides). This could introduce `horizontal pleiotropy'^9^ and lead to violation of assumption (ii) or (iii) due to variants affecting the outcome via a different biological pathway. This could in turn lead to bias, type I and type II error inflation, if unaccounted for. MR-Egger regression^10^ is a recently proposed method to both detect and adjust for pleiotropy in an MR-analysis.

Like all IV methods, the IVW approach is known to be vulnerable to weak instrument bias, which can be quantified for each genetic variant included in the analysis via its F-statistic.^11^ However, the F-statistic is not a sufficient indicator of instrument strength for MR-Egger. In this paper we clarify that instrument strength has a very different meaning for MR-Egger in the two-sample summary data context; it is a collective property of all genetic variants included in the analysis, and a `weak set' of instruments can be understood as inducing regression dilution bias^12^^,^^13^ into its estimate of causal effect. We formalize the cause of this dilution by defining it as a violation of the `NO Measurement Error' (NOME) assumption. The $I^{2}$ statistic^14^ is proposed to quantify the strength of NOME violation for a set of instruments used for MR-Egger regression, and the expected magnitude of regression dilution that will occur. We also describe how the established method of simulation extrapolation (SIMEX)^15^^,^^16^ can be used for bias-adjusted inference.

In the Methods section, we review the IVW and MR-Egger regression approaches to Mendelian randomization in the two-sample summary data context. We then explain the consequences of NOME violation for both methods for several hypothetical but general scenarios. In the Results section, we first show the impact of NOME violation on MR-Egger causal estimates using simulated data and the performance of bias adjustment via SIMEX. We then demonstrate our methods for a real two-sample summary data MR analysis on the effect of low-density lipoprotein and heart disease, using summary data from the Global Lipids Genetics and CARDIoGRAM consortia.^2^^,^^3^ We conclude with a discussion of the issues raised and point to future research.

Technical details are kept to a minimum in the main body of the paper; the interested reader is directed to the Appendix (available as Supplementary data at *IJE* online) for further clarification where appropriate. 

## Methods

### Modelling assumptions

We assume that normally distributed summary data estimates are available for the single nucleotide polymorphism (SNP)-exposure associations (${\hat{\gamma }}_{1}$,…,${\hat{\gamma }}_{L}$) and SNP-outcome associations (${\hat{\Gamma }}_{1}$,…,${\hat{\Gamma }}_{L}$) of L uncorrelated variants, and have been obtained in independent samples of non-overlapping participants for the purposes of a two-sample MR study. We allow the precision of these estimates to differ across variants (for example due to allele frequency), denoting the variance of the jth SNP-exposure association and SNP-outcome association as $\sigma_{Xj}^{2}$ and $\sigma_{Yj}^{2}$, respectively. We assume throughout that each variant is truly associated with the exposure [IV assumption (i) holds] so that the underlying SNP-exposure association parameters $\gamma_{1},\ldots ,\gamma_{L}$ are all non-zero. Furthermore, we assume that the genetic data have been coded so that SNP-exposure associations are all positive. Our models for the jth SNP-exposure and SNP-outcome associations are as follows:

(1) $${\hat{\gamma }}_{j}∼N(\gamma_{j},\sigma_{Xj}^{2}),\;{\hat{\Gamma }}_{j}∼N(\alpha_{j}+\beta \gamma_{j},\sigma_{Yj}^{2}).$$

Here β represents the true causal effect that we wish to estimate and $\alpha_{j}$ allows for the possibility that genetic variant j could affect the outcome via a separate molecular pathway from the exposure X. We refer to $\alpha_{j}$ as the *pleiotropic effect* of variant j. A more detailed description of the modelling assumptions is provided in the Appendix (available as Supplementary data at *IJE* online).

The ratio estimate for the causal effect derived from the jth variant only, ${\hat{\beta }}_{j}$, is equal to the SNP-outcome association divided by the SNP-exposure association, ${\hat{\Gamma }}_{j}/{\hat{\gamma }}_{j}$.^17^ If variant j is a valid IV, then it is a consistent estimate for the causal effect β. It is common practice to assume that the variance of the SNP-exposure association is negligible.^5–7^ We call this the NO Measurement Error (NOME) assumption. Taken at face value, NOME implies $\sigma_{Xj}^{2}$ = 0 and therefore the estimate ${\hat{\gamma }}_{j}$ is identical to the true value $\gamma_{j}$ for all j. Under the NOME assumption, the variance of the jth ratio estimate $var({\hat{\beta }}_{j})=\sigma_{Yj}^{2}/{\hat{\gamma }}_{j}^{2}$ because ${\hat{\gamma }}_{j}$ is treated as a constant. This is equivalent to only the first term from a full Taylor series expansion of $var({\hat{\beta }}_{j})$.

### The IVW approach

The inverse variance weighted (IVW) estimate, ${\hat{\beta }}_{IVW}$, is a weighted average of the ratio estimates ${\hat{\beta }}_{1},\ldots ,{\hat{\beta }}_{L}$. The IVW method (as originally proposed) assumes that all variants are valid IVs so that none of the genetic variants exhibit pleiotropy and hence $\alpha_{j}$ = 0 for all j. It is common practice to use inverse variance weights derived via NOME^5^^,^^7^ so that it has the form:

(2) $${\hat{\beta }}_{IVW}=\frac{\sum_{j=1}^{L}w_{j}{\hat{\beta }}_{j}}{\sum_{j=1}^{L}w_{j}},\;w_{j}={\hat{\gamma }}_{j}^{2}/\sigma_{Yj}^{2}.$$

The IVW estimate can be equivalently obtained as the slope from a linear regression of the SNP-outcome association estimates on the SNP-exposure association estimates with the intercept term constrained to zero.

If the above assumptions are satisfied, ${\hat{\beta }}_{IVW}$ is an unbiased estimate for β. However, NOME will never be perfectly satisfied in practice. In the presence of substantial measurement error, the IVW estimate is known to suffer from weak instrument bias,^18^ where instrument strength is typically represented by the F-statistic. In the two-sample summary data context and with uncorrelated genetic variants, the F-statistic for variant j can be approximated as $F_{j}$ = ${\hat{\gamma }}_{j}^{2}/\sigma_{Xj}^{2}$. We use this approximation for the remainder of the paper. In the two-sample context considered here, the effect of weak instrument bias is to attenuate the causal effect towards the null.^4^

### MR-Egger regression

In contrast to the IVW method, MR-Egger regression^10^ does not assume that all of the SNP-outcome associations are unaffected by pleiotropy, so the $\alpha_{j}$s are all allowed to be non-zero. Put simply, it assumes that the magnitude of the pleiotropic effects are independent of their strengths as instruments. That is, the size of $\gamma_{j}$ for variant j provides no information as to the size of its corresponding $\alpha_{j}$. This is referred to as the InSIDE (Instrument Strength Independent of Direct Effect) assumption;^10^ see also the Appendix for a more detailed discussion (available as Supplementary data at *IJE* online). In common with the IVW method, MR-Egger makes the NOME assumption. It performs a regression of the SNP-outcome associations on the SNP-exposure association of the form ${\hat{\Gamma }}_{j}=\beta_{0E}+\beta_{1E}{\hat{\gamma }}_{j}$. Weighting the regression by the precision of ${\hat{\Gamma }}_{j}$ improves efficiency and is recommended, but for simplicity of explanation, we ignore this extra complication for now.

The intercept estimate ${\hat{\beta }}_{0E}$ can be interpreted as the average pleiotropic effect across all variants and the slope estimate ${\hat{\beta }}_{1E}$ provides an estimate for the true causal parameter β. MR-Egger can only detect pleiotropy when it is `directional' (i.e. it has a non-zero average value), since only then will $\beta_{0E}$ be non-zero. It could be, for example, that all variants exhibit pleiotropy, but on average it cancels out. This is referred to as `balanced' pleiotropy.^10^ When InSIDE and NOME are perfectly satisfied, MR-Egger returns an unbiased estimate for the causal effect β. However, when InSIDE holds but NOME is violated, it will not be unbiased; its expected value will equal the true value β multiplied by a scale factor between 0 and 1, as below:

Expected value of MR-Egger slope:

(3) $${\hat{\beta }}_{\text{1E}}\approx \beta \frac{Var(\gamma )}{Var(\hat{\gamma })}\text{=}\beta \frac{\sigma_{\gamma }^{\text{2}}}{\sigma_{\gamma }^{\text{2}}{\text{+s}}^{\text{2}}}.$$

Here, $\sigma_{\gamma }^{2}$ is the variance of the set of true SNP-exposure associations $\gamma_{1},\ldots ,\gamma_{L}$ and $s^{2}$ represents the additional `average' variability among ${\hat{\gamma }}_{1},\ldots ,{\hat{\gamma }}_{L}$ due to estimation (or measurement) error. Only when $s^{2}$ is zero will NOME be satisfied. When the SNP-exposure estimates are more variable than the underlying parameter values, so that $s^{2}$ is non-zero, the resulting NOME violation leads to the MR-Egger estimate being attenuated towards zero, as per formula (3). This attenuation can be understood as an archetypal case of regression dilution bias. A more detailed explanation of formula (3) is given in the Appendix (available as Supplementary data at *IJE* online).

### Assessing regression dilution with $I_{GX}^{2}$

The ratio $\text{var}(\gamma )/\text{var}(\hat{\gamma })$ in equation (3), and hence the magnitude of the regression dilution, can be approximated using the $I^{2}$ statistic,^14^ a well-known tool for assessing between study heterogeneity in meta-analysis. First, define Cochran's *Q* statistic for the SNP-exposure associations to be:

$$Q_{GX}=\sum_{j=1}^{L}\frac{{\left({\hat{\gamma }}_{j}-\bar{\hat{\gamma }}\right)}^{2}}{\sigma_{Xj}^{2}}$$

where $\bar{\hat{\gamma }}$ is the mean of the SNP-exposure associations (weighted by 1/$\sigma_{Xj}^{2}$). Our corresponding $I^{2}$ statistic is then defined to be $I_{GX}^{2}$ = $(Q_{GX}-(L-1))/Q_{GX}$. Our approximation implies that, on average, ${\hat{\beta }}_{1E}$ is roughly equal to $\beta I_{GX}^{2}$, as suggested by formula (3); see the Appendix for further details and the slightly adapted formula for $I_{GX}^{2}$ under a weighted analysis, available as Supplementary data at *IJE* online.

**Figure 1.**
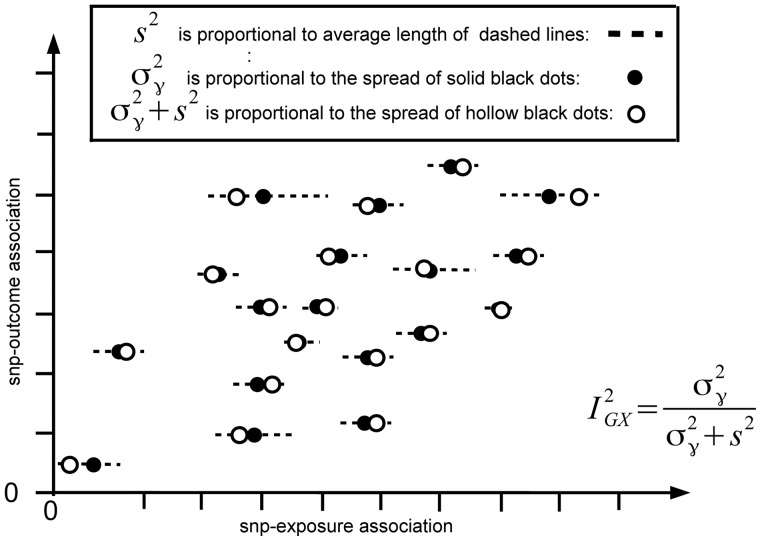
Illustrative diagram showing the SNP-exposure associations (estimates = hollow black dots, true values = solid black dots) plotted against the SNP-outcome association estimates for a fictional MR analysis.

In order to clarify the definition of $I_{GX}^{2}$ in the MR context, we refer to Figure 1. The solid black dots show a scatter plot of the SNP-outcome association estimates (the $\hat{\Gamma }$s) versus the true SNP-exposure associations (the γs). The hollow black dots show the SNP-outcome association estimates versus the SNP-exposure association estimates (the $\hat{\gamma }$s). In practice, we only observe the hollow black dots (estimate versus estimate). The horizontal dashed lines centred at each solid black dot represents the region within which we would expect to find the estimate ${\hat{\gamma }}_{j}$ (hollow black dot) with 95% probability, given that it is generated from equation (1). These lines are proportional in length to the standard error of each SNP-exposure association estimate. Note that there is variation in the length of the dashed lines, because each SNP-exposure standard error is unique, depending on factors such as allele frequency. The $I_{GX}^{2}$ statistic represents the true variance of the SNP-exposure associations, $\sigma_{\gamma }^{2}$ (or spread of black dots) divided by the variance of the SNP-exposure association estimates, $\sigma_{\gamma }^{2}+s^{2}$ (or spread of hollow black dots).

The $I_{GX}^{2}$ statistic therefore offers a convenient interpretation. When the underlying SNP-exposure associations are sufficiently heterogeneous, and the uncertainty in the SNP-exposure association estimates is small in comparison with this underlying variability, $I_{GX}^{2}$ will be close to 1 and the attenuation due to NOME violation will be negligible. If the underlying associations are generally similar in magnitude, or if their estimates are relatively imprecise (or both), then $I_{GX}^{2}$ could be much less than 1 and the attenuation will be severe. An $I_{GX}^{2}$ statistic of 0.9 provides the assurance that the likely bias in ${\hat{\beta }}_{1E}$ due to measurement error is around 10% of the true value of β. This is, equivalent to the assurance provided by an F-statistic of 10 in traditional IV analyses.

### The impact of regression dilution: further examples

**Figure 2.**
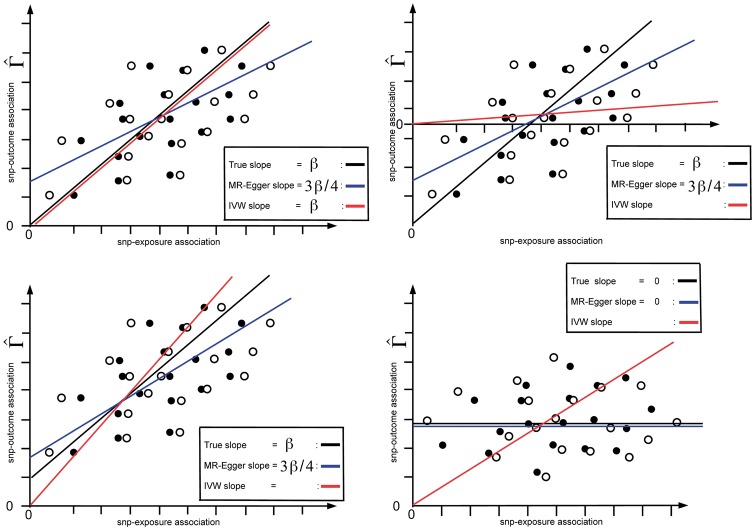
Illustrative diagram showing the SNP-outcome association estimates plotted against both the SNP-exposure association estimates (hollow black dots) and their true values (solid black dots). Top left: positive causal effect, balanced pleiotropy. Top right: positive causal effect, negative directional pleiotropy. Bottom left: positive causal effect, positive directional pleiotropy. Bottom right: no causal effect, positive directional pleiotropy.

In order to gain further insight into the impact of regression dilution for MR-Egger, consider the scatter plots of hypothetical summary data shown in Figure 2. Here we have removed error bars indicating the uncertainty in the SNP-exposure association estimates for clarity. In each scatter plot, InSIDE is assumed to hold.

In Figure 2 (top left), we imagine that all variants are invalid instruments but the pleiotropy is balanced. That is, $\alpha_{1},\ldots ,\alpha_{L}$ are all non-zero but their average value is zero. Furthermore, the causal effect β is positive. We also assume that all the variants are strong instruments in the traditional sense of having large F-statistics, but that NOME is violated to the extent that $I_{GX}^{2}$ = 0.75. As before, each hollow black dot represents (${\hat{\gamma }}_{j},{\hat{\Gamma }}_{j}$) [the SNP-exposure association estimates versus the SNP-outcome association estimates] whereas the solid black dots show the true $\gamma_{j}$s plotted against the ${\hat{\Gamma }}_{j}$s. Note again that the hollow black dots are more variable than the solid black dots.

Since all instruments are strong and the pleiotropy is balanced ($\beta_{0E}$ = 0), the IVW estimate perfectly aligns with the true slope, which is denoted by the solid black line. However, because NOME is violated, we expect the MR-Egger estimate to be diluted towards zero by a factor of $I_{GX}^{2}$ = 3/4. This is shown by the solid blue line. Since the slope and intercept parameter estimates from MR-Egger are negatively correlated, this means that the intercept parameter estimate is positively biased. In the Results section, we show that this leads to an inflation in the type I error rate of the MR-Egger test for directional pleiotropy.

Figure 2 (top right) shows the same scenario as Figure 2 (top left) except instead of balanced pleiotropy, there is now negative directional pleiotropy. As before, the MR-Egger slope parameter is diluted towards zero from β by a factor of 3/4. In this case the intercept estimate is also attenuated, meaning that the power of the MR-Egger test to detect true directional pleiotropy is reduced. In this example the IVW estimate (shown by the red line) is much closer to the null, due to the pleiotropy acting in the opposite direction of the causal effect. Its bias is solely due to the incorrect assumption that all variants are valid IVs, and not because of regression dilution. Figure 2 (bottom left) shows the case where there is positive directional pleiotropy and a positive causal effect. In this example the IVW estimate is further from the null, since the pleiotropy acts in the same direction as the causal effect. The MR-Egger slope parameter is diluted towards zero as before but the intercept estimate is increased. However, since it is truly non-zero, this does not lead to type I error inflation. Figure 2 (bottom right) shows the case where there is positive directional pleiotropy, but the causal effect is zero. In this case, violation of the NOME assumption has no effect; if the causal effect is truly zero then it cannot be attenuated any further. As in Figure 2 (bottom left), the IVW method mistakenly attributes the positive pleiotropy to a causal effect.

### Bias adjustment via simulation extrapolation

**Figure 3.**
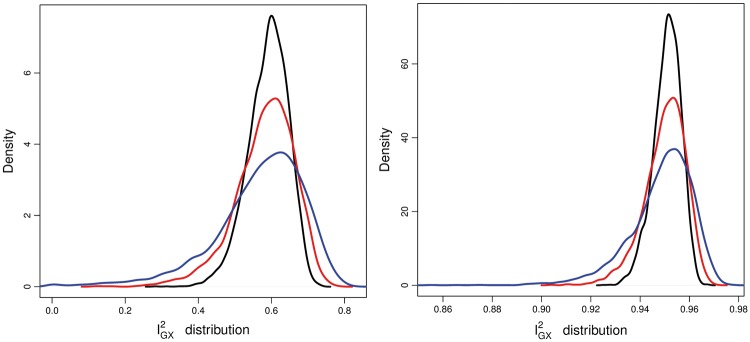
Left: distribution of $I_{GX}^{2}$ estimates under scenario 1 for $\bar{F}$ = 20 and $I_{GX}^{2}$ = 0.60 when L = 25 (blue), 50 (red) and 100 (black). Right: distribution of $I_{GX}^{2}$ estimates under scenario 1 for $\bar{F}$ = 125 and $I_{GX}^{2}$ = 0.95 when L = 25 (blue), 50 (red) and 100 (black).

An intuitive but crude bias-corrected estimate for the causal effect would be ${\hat{\beta }}_{1E}/I_{GX}^{2}$. In a preliminary investigation, however, this approach did not work well. Even when the true value of $I_{GX}^{2}$ is large, its estimate is a random quantity and can sometimes be close or equal to zero (as we will subsequently illustrate and explain in Figure 3). Therefore, simply dividing the original MR-Egger estimate by $I_{GX}^{2}$ can yield unstable results. We found that the well-established technique of simulation extrapolation (SIMEX)^15^ to be more reliable. Under SIMEX, new data sets are created by simulating SNP-exposure association estimates under increasing violations of the NOME assumption. That is, for each new data set, a new SNP-exposure estimate for variant j is generated with a mean value equal to the observed estimate $\left({\hat{\gamma }}_{j}\right)$, but with a variance that is (1+λ) times as large as $\sigma_{Xj}^{2}$ (where λ is a non-negative number). The simulated data are combined with the observed SNP-outcome estimates to yield a new value for ${\hat{\beta }}_{1E}$. This is repeated many times for the same value of λ to get an average value for ${\hat{\beta }}_{1E}$, and the whole process is repeated for a range of λs. The average value of ${\hat{\beta }}_{1E}$ tends to get smaller as the magnitude of λ increases, since the regression dilution effect will be stronger. A statistical model is then fitted to the set of average values obtained across the λs, by treating them as data points. The fitted model then enables the user to extrapolate back and estimate the value of ${\hat{\beta }}_{1E}$ that would have been obtained if NOME had been satisfied. This can be viewed conceptually as setting λ to -1 to perfectly remove the measurement error: see Figure 5 for an illustration of the method in practice and the Appendix for further technical details (available as Supplementary data at *IJE* online). R and Stata packages exist to implement SIMEX^16^^,^^19^, providing point estimates as well as accompanying standard errors to enable full inference after bias adjustment.

## Results

### Simulations

In this section we demonstrate the impact of NOME violation (as measured by $I_{GX}^{2}$) on the performance of MR-Egger regression and the IVW approach, and show the utility of bias adjustment for MR-Egger regression via SIMEX. Data sets of 25 SNP-exposure and SNP-outcome associations were generated to furnish two-sample summary data MR analyses in the following manner.

#### SNP-exposure associations for a given $\bar{F}$ and $I_{GX}^{2}$

SNP-exposure standard errors $\sigma_{X1}$,…,$\sigma_{X25}$ and SNP-exposure parameters $\gamma_{1},\ldots ,\gamma_{25}$ were drawn from Uniform distributions and then used to generate SNP-exposure estimates ${\hat{\gamma }}_{1},\ldots ,{\hat{\gamma }}_{25}$ from model (1). The lower and upper bounds of these distributions were chosen in order to fix the true mean F-statistic ($\bar{F}$) and $I_{GX}^{2}$ to the specific values desired. Initially, we fix $\bar{F}$ to be close to that of the lipids data analysed in the following section ($\bar{F}$ = 125) and then consider four values of $I_{GX}^{2}$: 95%, 90%, 85% and 75%. Both Table 4 and Figure 7 (left) in the Appendix (available as Supplementary data at *IJE* online) show, for each value of $I_{GX}^{2}$, the precise sampling distributions for $\sigma_{X1}^{2}$,…,$\sigma_{X25}^{2}$, $\gamma_{1},\ldots ,\gamma_{25}$ and the resulting distribution of F-statistics (with mean value 125). By letting $\sigma_{X1}^{2}$,…,$\sigma_{X25}^{2}$ take a range of values, we can account for heterogeneity in the precisions of SNP-exposure estimates present in real data due to differing allele frequency.

#### SNP-outcome associations for a given pattern of pleiotropy

SNP-outcome standard errors were generated by setting $\sigma_{Yj}$ equal to $2*\sigma_{Xj}$ in order to reflect a common allele frequency for a given variant j but different sample sizes in the underlying SNP-exposure and SNP-outcome cohorts. Pleiotropy parameters $\alpha_{1},\ldots ,\alpha_{25}$ were randomly generated from a Uniform distribution under five distinct scenarios. For a fixed causal effect, β, $\alpha_{1},\ldots ,\alpha_{25}$ and $\sigma_{Y1},\ldots ,\sigma_{Y25}$ were then used to generate SNP-outcome association estimates ${\hat{\Gamma }}_{1},\ldots ,{\hat{\Gamma }}_{25}$ from model (1). The five simulation scenarios explored were:

- Scenario 1: balanced pleiotropy [$\alpha_{j}∼$ Uniform(-0.2,0.2)] and a positive causal effect: consistent with $\beta =\beta_{1E}=1,$$\beta_{0E}=0;$
- Scenario 2: negative directional pleiotropy [$\alpha_{j}∼$ Uniform(-0.2,0)] and a positive causal effect: consistent with $\beta =\beta_{1E}=1,$$\beta_{0E}=-0.1;$
- Scenario 3: positive directional pleiotropy [$\alpha_{j}∼$ Uniform(0,0.2)] and a positive causal effect: consistent with $\beta =\beta_{1E}=1,$$\beta_{0E}=0.1;$
- Scenario 4: positive directional pleiotropy [$\alpha_{j}∼$ Uniform(0,0.2)] and a zero causal effect: consistent with $\beta =\beta_{1E}=0,$$\beta_{0E}=0.1;$
- Scenario 5: no pleiotropy [all $\alpha_{j}$ = 0] and a zero causal effect: consistent with $\beta =\beta_{1E}=0,$$\beta_{0E}=0.$

Note that in each scenario, the MR-Egger intercept parameter $\beta_{0E}$ is equal to the arithmetic mean of the pleiotropy parameter distribution. Scenarios 1, 2, 3 and 4 mirror the situations highlighted in Figure 2 (top-left, top-right, bottom-left and bottom-right, respectively). The additional scenario (scenario 5) is strictly the only one where the assumptions underlying the IVW approach (as originally proposed) are satisfied.

An important facet of our simulations is that, for each summary data set, SNP-exposure and pleiotropy parameters (the γs and αs) are generated from independent distributions, as in reference (10). Following this procedure enables us to see how MR-Egger regression would work on average across different MR data sets of the same size, as opposed to a single data set with fixed parameter values. This means we avoid having to pick specific values for the γs and αs, which could be seen as arbitrary. It also guarantees that, across the simulations, the average correlation between instrument strength and pleiotropy parameters will be zero (so that InSIDE is satisfied `on average'). However, for any single data set, this correlation will be non-zero and InSIDE will be strictly violated. We therefore refer to this data-generating procedure as satisfying the `weak' InSIDE assumption, a concept which is further clarified in the Appendix, available as Supplementary data at *IJE* online.

Exploring all five scenarios under the four values of $I_{GX}^{2}$ gave 20 simulation settings in total. For the IVW approach, we report the average causal effect estimate ${\hat{\beta }}_{IVW}$ and the probability of rejecting the causal null hypothesis β = 0. For MR-Egger regression (with and without adjustment via SIMEX) we report: the average estimate for the intercept parameter ${\hat{\beta }}_{0E}$ and the probability of rejecting the null hypothesis of no directional pleiotropy ($\beta_{0E}=0$); and the average estimate of the slope parameter ${\hat{\beta }}_{1E}$ and the probability of rejecting the causal null (β = 0). All methods were implemented as described in the Results section, using t-tests for hypothesis testing at the 5% significance level. The results, which are the average of 5000 simulations, are shown in Table 1. We label the rejection probabilities as *P*: when the null hypothesis is true, *P* equals the type I error rate, and when the null hypothesis is false, *P* equals the power. The first two columns of Table 1 show the true value of $I_{GX}^{2}$ and its average estimated value (they are close but not exactly equal). The most striking single observation is that, across all simulation settings, the average unadjusted MR-Egger estimate of causal effect, ${\hat{\beta }}_{1E}$, is approximately equal to β times the average $I_{GX}^{2}$ estimate, in line with our theoretical prediction.

**Table 1.** Results for simulation scenarios 1–5, $\bar{F}$ = 125. P equals power for ${\hat{\beta }}_{1E}$ and ${\hat{\beta }}_{IVW}$ in scenarios 1, 2 and 3 and type I error in scenarios 4 and 5. P equals power for ${\hat{\beta }}_{0E}$ in scenarios 2, 3 and 4 and type I error in scenarios 1 and 5

			MR-Egger regression		
$I_{GX}^{2}$	IVW	Standard approach		SIMEX adjusted	
True Est	${\hat{\beta }}_{IVW}$(*P*)	${\hat{\beta }}_{0E}$(*P*)	${\hat{\beta }}_{1E}$(*P*)	${\hat{\beta }}_{0E}$(*P*)	${\hat{\beta }}_{1E}$(*P*)
	Scenario 1: Balanced pleiotropy, $\beta =\beta_{1E}$ = 1, $\beta_{0E}$ = 0				
0.95 0.95	0.99 (1.00)	0.02 (0.06)	0.95 (1.00)	0.00 (0.05)	1.00 (1.00)
0.90 0.90	0.99 (1.00)	0.04 (0.07)	0.89 (0.94)	0.00 (0.05)	0.99 (0.94)
0.85 0.84	0.99 (1.00)	0.07 (0.08)	0.84 (0.73)	0.01 (0.06)	0.98 (0.73)
0.75 0.73	0.99 (1.00)	0.11 (0.10)	0.73 (0.41)	0.02 (0.06)	0.95 (0.44)
	Scenario 2: Negative directional pleiotropy, $\beta =\beta_{1E}$ = 1, $\beta_{0E}$ = -0.1				
0.95 0.95	0.78 (1.00)	−0.08 (0.28)	0.95 (1.00)	−0.10 (0.38)	1.00 (1.00)
0.90 0.90	0.76 (1.00)	−0.06 (0.12)	0.89 (1.00)	−0.10 (0.20)	0.99 (1.00)
0.85 0.84	0.75 (1.00)	−0.03 (0.07)	0.84 (0.94)	−0.09 (0.14)	0.98 (0.94)
0.75 0.73	0.75 (1.00)	0.01 (0.05)	0.73 (0.69)	−0.08 (0.09)	0.94 (0.71)
	Scenario 3: Positive directional pleiotropy, $\beta =\beta_{1E}$ = 1, $\beta_{0E}$ = 0.1				
0.95 0.95	1.20 (1.00)	0.12 (0.56)	0.95 (1.00)	0.10 (0.39)	1.00 (1.00)
0.9 0.90	1.22 (1.00)	0.14 (0.46)	0.90 (1.00)	0.10 (0.24)	1.00 (1.00)
0.85 0.84	1.23 (1.00)	0.16 (0.41)	0.84 (0.94)	0.10 (0.19)	0.99 (0.94)
0.75 0.73	1.23 (1.00)	0.21 (0.41)	0.73 (0.68)	0.12 (0.16)	0.94 (0.69)
	Scenario 4: Positive directional pleiotropy, $\beta =\beta_{1E}$ = 0, $\beta_{0E}$ = 0.1				
0.95 0.95	0.21 (0.98)	0.10 (0.46)	0.00 (0.05)	0.10 (0.43)	0.00 (0.05)
0.90 0.90	0.23 (0.99)	0.10 (0.29)	0.00 (0.05)	0.10 (0.25)	0.00 (0.05)
0.85 0.84	0.24 (1.00)	0.10 (0.20)	0.00 (0.05)	0.10 (0.17)	0.00 (0.06)
0.75 0.73	0.24 (1.00)	0.10 (0.15)	−0.01 (0.05)	0.10 (0.12)	−0.01 (0.06)
	Scenario 5: No pleiotropy, $\beta =\beta_{1E}$ = 0, $\beta_{0E}$ = 0				
0.95 0.95	0.00 (0.06)	0.00 (0.04)	0.00 (0.05)	0.00 (0.05)	0.00 (0.05)
0.90 0.90	0.00 (0.05)	0.00 (0.05)	0.00 (0.05)	0.00 (0.05)	0.00 (0.05)
0.85 0.84	0.00 (0.06)	0.00 (0.05)	0.00 (0.05)	0.00 (0.05)	0.00 (0.05)
0.75 0.73	0.00 (0.05)	0.00 (0.05)	0.00 (0.05)	0.00 (0.06)	0.00 (0.06)

### IVW results

Columns 3–4 of Table 1 show the performance of the IVW method. Since the 25 instruments are very strong, as measured by $\bar{F}$, the IVW method has an almost 100% rejection rate of the causal null for scenarios 1–4 (which all contain pleiotropy). However, this is its type I error rate for scenario 4, since the causal null is true. The type I error rate of the IVW estimate is preserved at the 5% level under scenario 5, when no pleiotropy or causal effect exists. Across all scenarios, the average value of ${\hat{\beta }}_{IVW}$ is very insensitive to changes in $I_{GX}^{2}$. Under scenarios 1 and 5, it is approximately unbiased and out-performs MR-Egger. Under scenarios 2–4, it is biased by a consistent amount due to the presence of directional pleiotropy and is inferior to MR-Egger.

### MR-Egger results

Columns 5–8 of Table 1 show the performance of the standard MR-Egger method, and columns 9–12 show the performance of MR-Egger with SIMEX adjustment. Under scenario 1, there is balanced pleiotropy and a positive causal effect ($\beta_{0E}$ = 0, β=1). The results show that increasing NOME violation (decreasing $I_{GX}^{2}$) leads to type I error inflation of the MR-Egger test for pleiotropy above the 5% level, due to over-estimation of $\beta_{0E}$. When $I_{GX}^{2}$ = 75%, the type I error rate is over 10%. Over-estimation of $\beta_{0E}$ coincides with under-estimation of the causal effect for MR-Egger and a reduction in the power to reject the causal null. SIMEX is able to mitigate the bias in ${\hat{\beta }}_{1E}$ caused by measurement error, reduce the type I error rate of the MR-Egger test for pleiotropy and leave the power to detect a causal effect unchanged.

Under scenario 2, there is negative directional pleiotropy and a positive causal effect ($\beta_{0E}$ = -0.1, β=1). Increasing NOME violation (decreasing $I_{GX}^{2}$) has the effect of reducing the power to detect directional pleiotropy and the power to detect a causal effect for MR-Egger. By adjusting for bias in ${\hat{\beta }}_{1E}$, SIMEX is able to marginally increase the power to detect directional pleiotropy and leave the power to detect a causal effect unchanged. Under scenario 3, there is positive directional pleiotropy and a positive causal effect ($\beta_{0E}$ = 0.1, β=1). The results are broadly similar to scenario 1. However, since there is true directional pleiotropy, the power to detect it increases with increasing NOME violation for MR-Egger. Applying SIMEX to successfully correct for bias in ${\hat{\beta }}_{0E}$ and ${\hat{\beta }}_{1E}$ then actually reduces the power to detect pleiotropy.

Under scenario 4, there is positive directional pleiotropy and a zero causal effect ($\beta_{0E}$ = 0.1, β=0). The results confirm that when the causal null hypothesis is true, the MR-Egger estimate ${\hat{\beta }}_{1E}$ is unbiased and consequently the type I error rate of the MR-Egger causal effect estimate is maintained at its nominal level. This is true regardless of the strength of NOME violation. Applying SIMEX in this case has no effect on inference for the causal effect, but slightly reduces the power to detect directional pleiotropy. Under scenario 5 there is no pleiotropy, and a zero causal effect ($\beta_{0E}$ = 0, β=0). In this case, the IVW method and MR-Egger (with and without SIMEX) all unbiasedly estimate their model parameters and their tests maintain the correct type I error rate.

### Further results for $\bar{F}$ = 20

Table 2 shows the results for a near identical simulation study, except that the mean instrument strength $\bar{F}$ is fixed at 20 and the true $I_{GX}^{2}$ values are varied between 60% and 40%. The strength and effect of the NOME violation are now much more severe.

**Table 2.** Results for simulation scenarios 1–5. $\bar{F}$ = 20. P equals power for ${\hat{\beta }}_{1E}$ and ${\hat{\beta }}_{IVW}$ in scenarios 1, 2 and 3 and type I error in scenarios 4 and 5. P equals power for ${\hat{\beta }}_{0E}$ in scenarios 2, 3 and 4 and type I error in scenarios 1 and 5

			MR-Egger regression		
$I_{GX}^{2}$	IVW	Standard approach		SIMEX adjusted	
True Est	${\hat{\beta }}_{IVW}$(*P*)	${\hat{\beta }}_{0E}$(*P*)	${\hat{\beta }}_{1E}$(*P*)	${\hat{\beta }}_{0E}$(*P*)	${\hat{\beta }}_{1E}$(*P*)
	Scenario 1: Balanced pleiotropy, $\beta =\beta_{1E}$ = 1, $\beta_{0E}$ = 0				
0.60 0.56	0.95 (1.00)	0.18 (0.22)	0.56 (0.38)	0.08 (0.10)	0.81 (0.41)
0.50 0.47	0.94 (1.00)	0.21 (0.28)	0.47 (0.24)	0.11 (0.12)	0.71 (0.29)
0.40 0.35	0.94 (1.00)	0.26 (0.33)	0.35 (0.14)	0.17 (0.15)	0.57 (0.19)
	Scenario 2: Negative directional pleiotropy, $\beta =\beta_{1E}$ = 1, $\beta_{0E}$ = -0.1				
0.60 0.56	0.73 (1.00)	0.08 (0.09)	0.56 (0.42)	−0.03 (0.08)	0.81 (0.46)
0.50 0.47	0.72 (1.00)	0.11 (0.13)	0.47 (0.29)	0.01 (0.09)	0.72 (0.34)
0.40 0.36	0.71 (1.00)	0.16 (0.19)	0.35 (0.16)	0.07 (0.10)	0.57 (0.21)
	Scenario 3: Positive directional pleiotropy, $\beta =\beta_{1E}$ = 1, $\beta_{0E}$ = 0.1				
0.60 0.56	1.17 (1.00)	0.28 (0.54)	0.56 (0.42)	0.18 (0.22)	0.81 (0.46)
0.50 0.47	1.17 (1.00)	0.31 (0.56)	0.47 (0.28)	0.21 (0.24)	0.71 (0.33)
0.40 0.35	1.17 (1.00)	0.35 (0.62)	0.37 (0.18)	0.26 (0.28)	0.59 (0.24)
	Scenario 4: Positive directional pleiotropy, $\beta =\beta_{1E}$ = 0, $\beta_{0E}$ = 0.1				
0.60 0.56	0.22 (0.54)	0.10 (0.12)	0.00 (0.05)	0.10 (0.11)	0.00 (0.07)
0.50 0.46	0.23 (0.56)	0.10 (0.12)	0.01 (0.06)	0.10 (0.11)	0.01 (0.08)
0.40 0.35	0.23 (0.57)	0.10 (0.11)	0.00 (0.06)	0.10 (0.12)	−0.01 (0.09)
	Scenario 5: No pleiotropy, $\beta =\beta_{1E}$ = 0, $\beta_{0E}$ = 0				
0.60 0.56	0.00 (0.05)	0.00 (0.05)	0.00 (0.06)	0.00 (0.07)	0.00 (0.07)
0.50 0.46	0.00 (0.05)	0.00 (0.06)	0.00 (0.06)	0.00 (0.09)	0.00 (0.08)
0.40 0.36	0.00 (0.06)	0.00 (0.05)	0.00 (0.06)	0.00 (0.09)	0.00 (0.09)

Both Table 4 and Figure 7 (right) in the Appendix (available as Supplementary data at *IJE* online) show, for each value of $I_{GX}^{2}$, the precise sampling distributions for $\sigma_{X1}$,…,$\sigma_{X25}$, $\gamma_{1},\ldots ,\gamma_{25}$ and the resulting distribution of F-statistics (with mean value 20). Higher values of $I_{GX}^{2}$ are not mathematically possible when $\bar{F}$ is low, without letting the strength of individual genetic variants get unnaturally close to zero [in the sense that they would not be chosen as instruments in the first place due to violation of IV assumption (i)].

Across all simulations settings, the average estimated value of $I_{GX}^{2}$ (column 2) multiplied by the causal effect β still perfectly predicts the average MR-Egger causal estimate ${\hat{\beta }}_{1E}$ (column 8). However, SIMEX adjustment is less effective in correcting the MR-Egger parameters for bias when a causal effect exists (scenarios 1–3). In scenarios 4 and 5, where the causal null is true, unadjusted MR-Egger estimates are well behaved with the correct type I error rate, whereas SIMEX adjustment slightly increases the type I error rate above the nominal level. Thus, under the causal null and when $\bar{F}$ and $I_{GX}^{2}$ are low, bias adjustment can actually be worse than no adjustment at all. 

### Estimation of $I_{GX}^{2}$ as a function of $\bar{F}$ and L

The $I_{GX}^{2}$ statistic is estimated from the data, and is therefore subject to error. Its variability will be affected by the strength of the instruments (as measured by F) and the total number of instruments, L. Figure 3 (left) shows the distribution of $I_{GX}^{2}$ estimates under scenario 1 when $\bar{F}$ = 20, the true value of $I_{GX}^{2}$ = 0.6 and when L is 25, 50 and 100. Figure 3 (right) shows the distribution of $I_{GX}^{2}$ estimates under scenario 1 when $\bar{F}$ = 125, the true value of $I_{GX}^{2}$ = 0.95 and when L is 25, 50 and 100. In both plots it is apparent that, as L increases, the variability in $I_{GX}^{2}$ estimates decreases and its distribution becomes less skewed. Crucially, for the $\bar{F}$ = 20 case, increasing L removes the possibility of estimating $I_{GX}^{2}$ to be zero. This clarifies why the crude adjustment for NOME violation (dividing ${\hat{\beta }}_{1E}$ by the estimated $I_{GX}^{2}$) can fail when $\bar{F}$ and L are low. On the contrary, we see in Figure 3 (right) that, when $\bar{F}$, L and $I_{GX}^{2}$ are high, the variability in estimated $I_{GX}^{2}$ is sufficiently small for crude bias adjustment to work well.

### Assessing the causal effect of LDL-c on CAD

There is a long and extensive literature on the association between various lipid fractions and coronary artery disease (CAD), but still far from universal agreement as to whether all these associations have a causal basis. We focus on the possible role of the least controversial lipid, low-density lipoprotein cholesterol (LDL-c), in modifying CAD risk. Using summary data from the Global Lipids Genetics Consortium (GLGC)^3^ to provide SNP-LDL-c association estimates, and summary data from CARDIoGRAM^2^ to provide SNP-CAD association estimates, we perform a two-sample MR analysis to illustrate the utility of MR-Egger regression and the $I_{GX}^{2}$ statistic.

Since LDL-c levels are closely related and highly correlated with other lipid fractions, we selected the 57 variants that were more strongly associated with LDL-c than with triglycerides or high density lipoprotein. This strategy (although not foolproof) aimed to reduce the possibility that, if pleiotropy existed among the variants, it is operating via a confounder of LDL-c and CAD. This would violate IV assumption (ii) and lead to violation of InSIDE. This would in turn bias the results from MR-Egger regression (regardless of the value of $I_{GX}^{2}$) as explored in reference (10). The minimum *P*-value for the strength of association across all variants was 8.3×10${}^{-7}$. The mean F-statistic across all included variants was 132, the weakest being 30 and the strongest being 1325.

**Figure 4.**
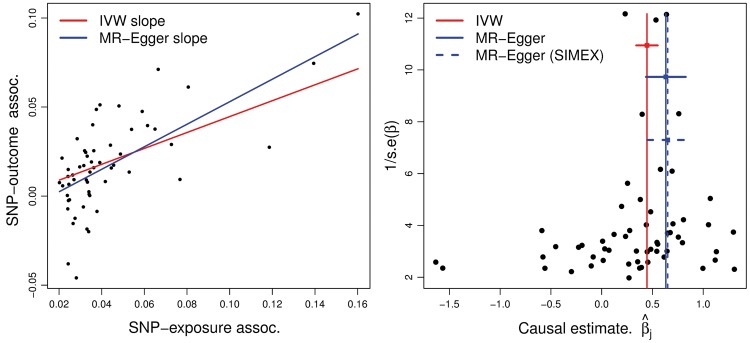
Left: scatter plot of the summary data estimates, with IVW and MR-Egger slope estimates shown. Right: funnel plot of the causal effect estimates, with overall estimates under the IVW and MR-Egger approaches (with and without SIMEX correction).

Figure 4 (left) shows a scatter plot of the SNP-outcome log-odds ratio associations (${\hat{\Gamma }}_{j}$) versus the SNP-exposure associations (${\hat{\gamma }}_{j}$) across all 57 included variants. The data are scaled so that the causal effect estimates represent log-odds ratios of CAD for a standard deviation increase in LDL-c. Figure 4 (right) shows a funnel plot^20^ of the causal effect estimates ${\hat{\beta }}_{j}$ = ${\hat{\Gamma }}_{j}/{\hat{\gamma }}_{j}$ on the x-axis versus their inverse standard error (a measure of their strength as instruments) on the y-axis. The funnel representation is a convenient tool for assessing the presence of directional pleiotropy. This would induce a correlation between effect size and instrument strength, leading to asymmetry in the plot.^10^ The IVW method estimates a strong positive causal effect of 0.45. However, there is reason to believe this analysis to be misleading, given that some asymmetry exists. Applying MR-Egger regression using code provided in online supplementary material accompanying reference (10) (and weighting by the inverse standard error of the SNP-outcome association to improve efficiency), negative directional pleiotropy is detected, although the evidence is not particularly strong. Consequently, the point estimate for $\beta_{1E}$ is adjusted upward to 0.63.

**Figure 5.**
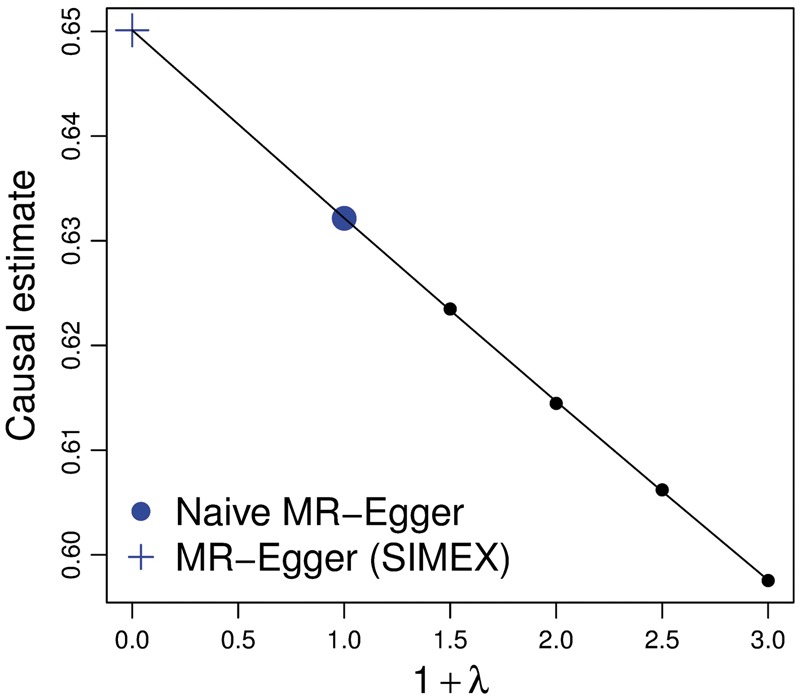
Simulation extrapolation applied to the MR-Egger regression analysis of the lipids data. The adjusted estimate is that predicted by the model at the value λ = -1.

We now assess the potential for regression dilution bias to attenuate the MR-Egger estimate for the causal effect. Under the weighted analysis considered here, $I_{GX}^{2}$ is calculated from $Q_{GX}$ using the weighted SNP-exposure associations and corresponding standard errors (${\hat{\gamma }}_{j}/\sigma_{Yj}$, $\sigma_{Xj}/\sigma_{Yj}$), whereas the unweighted analysis uses (${\hat{\gamma }}_{j}$, $\sigma_{Xj}$); see the Appendix for further details, available as Supplementary data at *IJE* online. For these data, $I_{GX}^{2}$ equals 0.971 for the standard weighted analysis and 0.974 for an unweighted analysis. A crude bias adjustment would therefore be 0.63/$I_{GX}^{2}$ = 0.65. We used the simex() package in R^19^ to implement the SIMEX method, choosing the quadratic model for the extrapolation (see Appendix). The SIMEX estimate was in very close to agreement with the crude bias-adjusted estimate of 0.65, as illustrated in Figure 5. Indeed, this is exactly what our simulations predicted given the observed values of $I_{GX}^{2}$, $\bar{F}$ and L (see Figure 3). Full results for all three methods are shown in Table 3. As in the supplementary material accompanying reference (10), causal effect estimates (Est), standard errors (SE), *P*-values and t-test values are calculated for each method under a multiplicative random effects model that accounts for over-dispersion (in this case due to pleiotropy).

**Table 3.** IVW and MR-Egger regression analysis (with and without SIMEX adjustment) of the lipids data

Model				
Parameter	Est	SE	t-value	p-value
IVW approach				
$\beta_{IVW}$	0.45	0.053	8.51	1.13e-11
MR-Egger regression				
$\beta_{0E}$	−0.0102	0.0046	−2.23	0.0298
$\beta_{1E}$	0.632	0.0975	6.481	2.66e-08
MR-Egger regression+SIMEX				
$\beta_{0E}$	−0.0109	0.0047	−2.33	0.0236
$\beta_{1E}$	0.6500	0.10000	6.47	2.76e-08

In conclusion, although borderline evidence of pleiotropy exists across the included variants, there is still strong evidence that LDL-c is causally related to CAD risk. MR-Egger regression revises the causal effect of LDL-c upwards, because the apparent causal effect is masked by pleiotropy acting in the opposing direction. Applying the SIMEX algorithm revises the estimate slightly further still, although a corresponding small increase in the standard errors leaves inference largely unchanged. We can at least confidently state that NOME violation is not a problem for these data.

It is of course perfectly possible that our strategy for including (and excluding) variants in the analysis could in fact be unduly influencing the results by inducing collider bias. We do not therefore claim that this approach is superior to the more liberal inclusion policy adopted by Holmes *et al*.^8^ in their main analysis, or that any single approach should be relied upon. Holmes *et al*. sensibly consider a range of analyses to address the problem of pleiotropy, for example by constructing both `restricted’ and `unrestricted’ gene scores. A spectrum of possible rules for including variants in an MR study are also discussed and implemented for very similar data by Bowden *et al*.^21^ Although we encourage such sensitivity analyses, they are beyond the scope of this paper.

## Discussion

The standard IVW method of Mendelian randomization with summary data makes the strong assumption that all variants are valid instruments, due to a complete absence of pleiotropy. However, if pleiotropy is present but balanced (as in Scenario 1 of the simulation study), it can still return unbiased estimates of causal effect and is considerably more powerful than MR-Egger regression. Unfortunately, the IVW method can give biased results under cases of directional pleiotropy and incorrectly infer causality (as in Scenario 4 of the simulations). MR-Egger regression, by contrast, is more robust to directional pleiotropy. It is not designed to replace the standard approach in the primary analysis, but is an important sensitivity analysis tool to probe whether the IV assumptions have been violated in a meaningful way.

Both the IVW and MR-Egger regression methods are traditionally implemented by assuming the SNP-exposure association is measured without error (the NOME assumption). Unfortunately, the price paid for MR-Egger's increased robustness to pleiotropy is a corresponding decrease in its robustness to violations of NOME, which manifests itself as regression dilution bias. Our work suggests that, in two-sample MR studies, the $I_{GX}^{2}$ statistic is a much more relevant summary measure for MR-Egger regression than the traditional F-statistic. Whereas the F-statistic is defined for each genetic variant and provides an independent assessment of its strength within an IVW analysis, $I_{GX}^{2}$ tells us that instrument strength is a singular, collective property of all variants within an MR-Egger analysis. Although better measures of instrument strength may still be developed for MR-Egger regression, the simplicity of $I_{GX}^{2}$ in calculation and interpretation make it an attractive option.

### Limitations and further work

Our focus in this paper was to explain the effect of violations to the NOME assumption on the performance of MR-Egger regression in the two-sample summary data context, and its connection to $I_{GX}^{2}$. Further work is required to understand the effect of NOME violation on MR-Egger regression when using a single-study population. The picture is likely to be more complex in this setting, since weak variants will induce bias towards the observational estimate, with the magnitude of the bias depending on the (unknown) strength of confounding. It may therefore be hard or impossible to find a statistic (like $I_{GX}^{2}$) to quantify this bias.

In order to make things as clear as possible, we purposefully simplified our two-sample MR data-generating model in several ways. First, data were simulated under the InSIDE assumption, so that we could be sure MR-Egger would return unbiased estimates when NOME is satisfied. If InSIDE is violated, for example due to pleiotropic effects acting via a confounder as explored in reference (10), an MR-Egger analysis would yield biased estimates even if $I_{GX}^{2}$ were equal to 1. In practice, for $I_{GX}^{2}$ values less than 1, its bias will likely be due to violations of InSIDE and NOME. Second, we generated summary SNP-exposure and SNP-outcome association estimates from independent normal distributions with known variance, rather than simulating the individual participant data directly, to furnish `idealised' two-sample MR analyses. This removed the two further issues of non-collapsibility and ascertainment bias which are often encountered in practice when the outcome is binary and case-control data are used to estimate log-odds ratios for the SNP-outcome associations.^22^ Extending methods such as MR-Egger regression to properly account for these issues is an important line of future research.

In this paper we showcased just one method of bias adjustment in the presence of measurement error, namely the SIMEX approach.^15^^,^^16^ We chose this because of its widespread use across statistics, its intuitive nature and its applicability to a wide range of statistical models. Software is also readily available to implement the approach in practice with very little computational burden. Of course, users may simply wish to implement a naive correction by dividing the observed MR-Egger causal estimate by $I_{GX}^{2}$. Although this will often be sufficient with a large number of strong instruments (as seen in the simulations and the lipids analysis) we do not think it is a reliable method in general. Furthermore, an estimate for the variance of the naive correction would also be needed to enable full statistical inference.

It is possible that alternative methods of bias adjustment could work better in the MR context, such as the plethora of approaches discussed in references^13^^,^^23^^,^^24^. For example, Sharp^25^ recommends a natural Bayesian formulation of the problem, where the bias issue can be circumvented by focusing directly on the parameters which subsequently generate the observed data affected by measurement error. However, it is worth noting that, by virtue of being shrunk towards zero, uncorrected estimates tend to have a smaller variance. Viewed through this lens, bias adjustment can then be seen as applying the appropriate correction factor to `reverse' the shrinkage, but at the cost of a reduced precision.

Model selection techniques have recently been proposed for MR analysis with the purpose of detecting and adjusting for invalid instruments, using methods that assume at least half of the genetic variants are valid instruments.^21^^,^^26^^,^^27^ MR-Egger regression can work even if all variants are invalid (under InSIDE), but our work has shown that its performance will be best when the $I_{GX}^{2}$ statistic is large. An obvious follow-on question, therefore, is whether it is sensible to adopt a strategy to increase the value of $I_{GX}^{2}$ for the analysis at hand. For example, it would be possible to combine variants together into a number of separate allele scores and to perform MR-Egger regression on them instead. The SNP-exposure estimates obtained from the individual allele scores would be smaller in number but more precise than those based on the individual variants, and could therefore give rise to a higher $I_{GX}^{2}$, as desired. These ideas naturally complement allele score approaches that have been shown to successfully mitigate weak instrument bias when performing standard two-stage least squares or IVW analyses.^18^^,^^28^

In conclusion, assessing the strength of NOME violation is an important prerequisite to performing causal inference with summary data, especially with MR-Egger regression. It is unfortunate that this fact was not clarified in the original publication by Bowden *et al*.,^10^ and we suspect the data examples contained in this paper would benefit from a more considered analysis in light of our increased understanding. It is comforting to note that standard MR-Egger regression remains a reliable method for testing the causal null hypothesis, even when NOME is violated. We recommend evaluating the $I_{GX}^{2}$ statistic alongside an MR-Egger analysis. If it is sufficiently low (less than 90%), point estimates of causal effect should be interpreted with caution due to regression dilution, and adjustment methods such as SIMEX should be considered as part of a sensitivity analysis.

## Supplementary Data

Supplementary data are available at *IJE* online.

## Funding

Jack Bowden is supported by an MRC Methodology Research Fellowship (grant MR/N501906/1). George Davey Smith is supported by the MRC Integrative Epidemiology Unit at the University of Bristol (grant code MC_UU_12013/1).

Key Messages

- MR-Egger regression provides a simple method for Mendelian randomization of summary data estimates that is robust to invalid instruments.
- MR-Egger regression is not designed to replace the standard approach as the primary analysis, but is an important sensitivity analysis to probe whether the IV assumptions have been violated in a meaningful way.
- MR-Egger assumes, like the standard implementation of the IVW method, that the variance of SNP-exposure association estimates is negligible (the NOME assumption).
- In the two-sample MR setting, if NOME is violated (as quantified by an $I_{GX}^{2}$ much less than 1) but InSIDE is satisfied, MR-Egger regression will tend to underestimate the causal effect and potentially inflate the type I error rate of the MR-Egger test for pleiotropy.
- An $I_{GX}^{2}$ value of 0.9 indicates a relative bias in the MR-Egger causal effect estimate of 10%. It provides an appropriate measure of instrument strength in the two-sample context.
- Like all statistics, $I_{GX}^{2}$ is an estimate: its variability will be affected by the strength of the instruments (as measured by the F-statistic) and the total number of instruments.
- Simulation extrapolation can be used to correct MR-Egger regression parameters for NOME violation, and in doing so reduce the type I error rate of the MR-Egger test for pleiotropy. Further research is required to find the optimal method of bias correction.
- NOME violation does not affect the type I error rate of the MR-Egger causal effect estimate.
- Further research is required to assess the effect of NOME violation in the single-sample MR context.

## Supplementary Material

Supplementary Data
