# Supplementary material for: Assessing the suitability of summary data for two-sample Mendelian randomization analyses using MR-Egger regression: the role of the I2 statistic
Source: Int J Epidemiol. 2016 Sep 11;45(6):1961–74. doi: 10.1093/ije/dyw220 (PMC5446088; doi:10.1093/ije/dyw220)
Supplement: Supplementary Data [file dyw220_supp.pdf]

Technical appendix to accompany "Assessing the suitability of summary data for two-sample Mendelian randomization analyses using MR-Egger regression: the role of the I^2 statistic"

Note: In this document we continue with the equation, figure and table numbering from the main body of the paper, but start a new numbering system for references.

Assume data on L uncorrelated genetic variants $G_1,...,G_L$, a continuous exposure X and outcome Y in the presence of a confounder U for a single sample of individuals. This is represented, for variant j, by the causal diagram in Figure 6. The relationships that are either required or forbidden by IV assumptions (i)-(iii) are labelled for clarity.

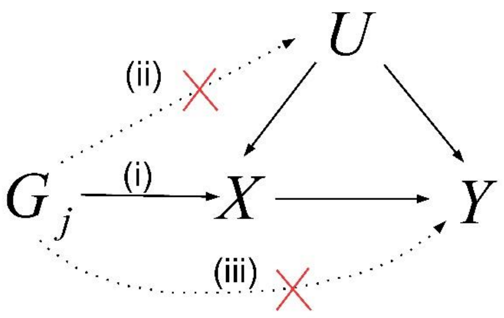

Figure 6: *Illustrative diagram relating genetic variant G_j to exposure X and outcome Y in the presence of a confounder U.*

Further assume the following underlying models linking G_j to X and Y for subject i:

$$X_i \mid G_{ij} = \gamma_0 + \gamma_j G_{ij} + \delta_{Xij}$$

$$Y_i \mid G_{ij} = \beta_0 + (\beta\gamma_j + \alpha_j)G_{ij} + \delta_{Yij}$$

$$= \beta_0 + \Gamma_j G_j + \delta_{Yij},$$

so that γ_j and Γ_j = ($\beta\gamma_j + \alpha_j$) represent G_j's association with the exposure and outcome respectively. We assume always that $\gamma_j \neq 0$ so IV assumption (i) holds. Let $\beta\gamma_j$ be the effect of G_j on Y through X, where β is the causal effect of X on Y we wish to estimate. The term α_j represents the association between G_j and Y not through the exposure of interest, thus violating IV assumptions (ii) or (iii).

When G, X and Y are collected on a single sample, the error terms δ_{Xij} and δ_{Yij} are correlated due to shared contributions from U. However, we now assume the same models can be applied to data collected from two independent populations, to furnish a two sample MR analysis. This means that δ_{Xij} and δ_{Yij} will be uncorrelated. With this in mind, we now move from an individual patient data model for G, X and Y, instead writing models for the L parameter estimates $\hat{\gamma}_j$ and $\hat{\Gamma}_j$ directly, in order to furnish a two-sample MR analysis using summary data.

$$\hat{\gamma}_j = \gamma_j + \epsilon_{Xj}, \quad \epsilon_{Xj} \sim N(0, \sigma^2_{Xj}) \tag{4}$$

$$\hat{\Gamma}_j = \alpha_j + \beta\gamma_j + \epsilon_{Yj}, \quad \epsilon_{Yj} \sim N(0, \sigma^2_{Yj}) \tag{5}$$

Models (4) and (5) are equivalent to model (1) in the main body of the paper. We assume that $(\epsilon_{Xj}, \epsilon_{Yj})$ are mutually independent and independent of γ_j. We also assume that σ^2_{Xj} and σ^2_{Yj} are known, not estimated. The InSIDE assumption states that the pleiotropic effect of each variant on the outcome is independent of its strength as an instrument for the exposure. For simplicity, we will now assume that InSIDE is **perfectly** satisfied in the model above. That is, the sample covariance of $\gamma_1,...,\gamma_L$ and $\alpha_1,....,\alpha_L$, denoted by $\text{cov}(\gamma_j, \alpha_j)$, is exactly zero.

Unbiased estimation of the IVW estimate under NOME

Suppose all variants are valid instruments, (α_j = 0 for all j). Assume also that the NOME assumption holds, so that σ^2_{Xj} = 0 for all j (or $\hat{\gamma}_j = \gamma_j$). The ratio estimate $\hat{\beta}_j = \hat{\Gamma}_j / \hat{\gamma}_j$ is an unbiased estimate for β, since

$$E[\hat{\Gamma}_j / \gamma_j] = E_i[\hat{\Gamma}_j] / \gamma_j = \frac{\beta\gamma_j}{\gamma_j} = \beta.$$

The IVW estimate given in (2) is therefore also unbiased since it is a weighted average of unbiased estimates, whose weights ($\gamma^2_j / \sigma^2_{Yj}$) are uncorrelated with the estimates themselves.

Unbiasedness of MR-Egger regression under NOME

The MR-Egger slope estimate $\hat{\beta}_{1E}$ (from an unweighted analysis for simplicity) is equal to the sample covariance of the SNP-outcome and SNP-exposure estimates, divided by the sample variance of the SNP-exposure estimates:

$$\hat{\beta}_{1E} = \frac{cov(\hat{\Gamma}_j, \hat{\gamma}_j)}{var(\hat{\gamma}_j)}.$$

Let $var(\gamma_j) = \sigma^2_\gamma$ be the sample variance of $\gamma_1,...,\gamma_L$. When NOME also holds this guarantees that $var(\hat{\gamma}_j)$ = $var(\gamma_j)$ = σ^2_γ. When InSIDE holds perfectly we have that

$$E[cov(\hat{\Gamma}_j, \hat{\gamma}_j)] = E[cov(\hat{\Gamma}_j, \gamma_j)] = \beta\text{var}(\gamma_j) = \beta\sigma^2_\gamma, \tag{6}$$

and therefore $E[\hat{\beta}_{1E}] = \beta\sigma^2_\gamma / \sigma^2_\gamma = \beta$.

Estimation for MR-Egger regression when NOME violated

We now consider the value for $\hat{\beta}_{1E}$ when InSIDE is perfectly satisfied but NOME is violated. We re-write the MR-Egger estimate as

$$\hat{\beta}_{1E} = \frac{cov(\hat{\Gamma},\hat{\gamma})}{var(\hat{\gamma})} = \frac{cov(\hat{\Gamma},\hat{\gamma})}{var(\gamma)}\frac{var(\gamma)}{var(\hat{\gamma})}$$

Taking expectations of this product, assuming that the two terms in the product are uncorrelated, and substituting the expectation of the ratio with a ratio of expectations, we get the approximate relation

$$E[\hat{\beta}_{1E}] = \beta E\left[\frac{var(\gamma)}{var(\hat{\gamma})}\right] \approx \beta \frac{\sigma_{\gamma}^2}{\sigma_{\gamma}^2 + s^2},$$

where s^2 is the arithmetic mean of the σ_{Xj}^2 s. This implies that, if σ_{γ}^2 were known, β could be crudely approximated as

$$\hat{\beta}_{1E}\frac{\sigma_{\gamma}^2 + s^2}{\sigma_{\gamma}^2}.$$

Direct adjustment for measurement error therefore requires, at the very least, an estimate for σ_{γ}^2. Many estimators of this quantity exist, see for example [1, 2, 3] . However, the I_{GX}^2 statistic provides a simple and convenient estimate of the total ratio $\sigma_{\gamma}^2 / (\sigma_{\gamma}^2 + s^2)$ instead. The I_{GX}^2 statistic is defined in this context as I_{GX}^2 = $(Q_{GX} - (L-1)) / Q_{GX}$, where

$$Q_{GX} = \sum_{j=1}^{L}\frac{(\hat{\gamma}_j - \bar{\hat{\gamma}})^2}{\sigma_{Xj}^2}, \quad \text{for} \quad \bar{\hat{\gamma}} = \frac{\sum_{j=1}^{L}\hat{\gamma}_j / \sigma_{Xj}^2}{\sum_{j=1}^{L} 1/\sigma_{Xj}^2}. \qquad (7)$$

Specifically, I_{GX}^2 is algebraically equivalent to $\sigma_{\gamma}^2 / (\sigma_{\gamma}^2 + s^2)$ when the following substitutions are made:

$$\sigma_{\gamma}^2 = \frac{(Q_{GX} - (L-1))}{\sum_{j=1}^{L}u_j - \frac{\sum_{j=1}^{L}u_j^2}{\sum_{j=1}^{L}u_j}}, \quad s^2 = \frac{(L-1)\sum_{j=1}^{L}u_j}{(\sum_{j=1}^{L}u_j)^2 - \sum_{j=1}^{L}u_j^2}, \quad \text{for} \quad u_j = 1/\sigma_{Xj}^2$$

Here, σ_{γ}^2 is the DerSimonian and Laird estimate [1] and when the u_j's are all equal, the substituted value of s^2 is equal to their arithmetic mean. I_{GX}^2 therefore provides a very simple, well known and accurate gauge as to the severity of the regression dilution problem.

I_{GX}^2 under a weighted analysis

For simplicity, in the paper and in the Appendix so far, we have assumed that the MR-Egger analysis is unweighted. However, Bowden et al [4] recommend weighting the regression to account for unequal variances across the SNP-outcome associations. This is equivalent to fitting the model

$$\hat{\Gamma}_j / \sigma_{Yj} = \alpha_j / \sigma_{Yj} + \beta\gamma_j / \sigma_{Yj} + \epsilon_{Yj} / \sigma_{Yj} \quad (8)$$

For the InSIDE assumption to be perfectly satisfied under a weighted analysis, the equivalent weighted sample covariance must be zero. The regression dilution we would expect to see under the weighted analysis can still be quantified by the I_{GX}^2 statistic, except we replace $\hat{\gamma}_j$ with $\hat{\gamma}_j / \sigma_{Yj}$ and σ_{Xj}^2 with $\sigma_{Xj}^2 / \sigma_{Yj}^2$ respectively when defining Cochran's Q statistic in equation (7).

Simulation Extrapolation

We used the SIMEX approach [5, 6] to produce bias adjusted estimates for the MR-Egger estimate $\hat{\beta}_{1E}$. Under the SIMEX approach, new data sets are created by simulating SNP-exposure association estimates under increasing violations of the NOME assumption. That is, for each new data set (and non-negative number λ), a new SNP-exposure estimate $\hat{\gamma}_j^{\lambda}$ is generated using the observed data $(\hat{\gamma}_j, \sigma_{Xj}^2)$ from the model:

$$\hat{\gamma}_j^{\lambda} \sim N(\hat{\gamma}_j, \lambda\sigma_{Xj}^2), \quad (9)$$

so that $var(\hat{\gamma}_j^{\lambda})$ = $(1+\lambda)\sigma_{Xj}^2$. Note that choosing λ = 0 simply returns the original estimate $\hat{\gamma}_j$ with known variance σ_{Xj}^2. For a given value of λ, we combine the simulated $\hat{\gamma}^{\lambda}$ s with the observed $\hat{\Gamma}$ s to estimate the average value of $\hat{\beta}_{1E}$, $\overline{\hat{\beta}_{1E}(\lambda)}$. The value of $\overline{\hat{\beta}_{1E}(\lambda)}$ will tend to get smaller as the magnitude of λ increases. A model is then fitted to the $\overline{\hat{\beta}_{1E}(\lambda)}$'s in order to extrapolate back to $\overline{\hat{\beta}_{1E}(-1)}$, to approximate the value that would have been obtained for $\hat{\beta}_{1E}$ if NOME had been satisfied. We used the simex() package in R [7] to implement the method, choosing the quadratic model for the extrapolation.

Generating data under the weak InSIDE assumption

So far in the Appendix we have assumed that the parameter values $\gamma_1,...,\gamma_L$ and $\alpha_1,....,\alpha_L$ are fixed quantities, and that their sample covariance is zero. In our simulations we actually generated summary data estimates $\hat{\gamma}_1,...,\hat{\gamma}_L$ and $\hat{\Gamma}_1,...,\hat{\Gamma}_L$ under models (4) and (5) by firstly simulating $\gamma_1,...,\gamma_L$ and $\alpha_1,....,\alpha_L$ from independent Uniform distributions. This meant that, for every specific realisation of $\gamma_1,...,\gamma_L$ and $\alpha_1,....,\alpha_L$, the sample covariance $cov(\gamma_j,\alpha_j)$ was close but not equal to zero, so that InSIDE was actually violated in the standard sense. However, the expected value of the sample covariance $cov(\alpha_j,\gamma_j)$ across all simulated pairs of $\gamma_1,...,\gamma_L$ and $\alpha_1,....,\alpha_L$ **was** equal to zero. That is:

$$E[cov(\alpha_j,\gamma_j)] = 0.$$

Thus, we refer to the above condition (and our simulations) as being under the *weak* InSIDE assumption. Simulating the data in this way enabled us to gauge how MR-Egger regression would work on average across different data sets of the same size. It also means that we avoided having to pick arbitrary values for the γ and α parameters, or values that forced $cov(\gamma_j, \alpha_j)$ to be exactly zero, which could be viewed as unrealistic.

R code

Calculating I^2_{GX}

We firstly give R code to calculate the I^2_{GX} statistic, using the function *Isq()* below

```
Isq        = function(y,s){
k          =  length(y)
w          = 1/s^2; sum.w  = sum(w)
mu.hat   = sum(y*w)/sum.w
Q          = sum(w*(y-mu.hat)^2)
Isq        = (Q - (k-1))/Q
Isq        = max(0,Isq)
return(Isq)
}
```

Assume that *BetaXG* and *seBetaXG* represent the vector of SNP-exposure estimates and standard errors, and *BetaYG* and *seBetaYG* represent the vector of SNP-exposure estimates and standard errors. We calculate the I^2_{GX} statistic for an unweighted MR-Egger analysis using Isq*( )* above as

```
Isq(BetaXG,seBetaXG)
```

When performing a weighted MR-Egger analysis, we calculate I^2_{GX} identically, except we replace $\hat{\gamma}_j$ with $\hat{\gamma}_j / \sigma_{Yj}$ and σ_{Xj} with $\sigma^2_{Xj} / \sigma_{Yj}$ in the formulae above:

```
Isq(BetaXG/seBetaYG,seBetaXG/seBetaYG)
```

R code to perform Simulation Extrapolation

R code to perform the IVW and MR-Egger approaches can be found in the on-line appendix of [4]. Additional code to perform Simulation Extrapolation and to plot the results can be found below

```
# load the simex package
library(simex)
# MR-Egger regression (weighted)
Fit2 = lm(BetaYG~BetaXG,weights=1/seBetaYG^2,x=TRUE,y=TRUE)
# Simulation extrapolation
mod.sim <- simex(Fit2,B=1000,
        measurement.error = seBetaXG,
        SIMEXvariable="BetaXG",fitting.method ="quad",asymptotic="FALSE")
# plot results
l = mod.sim$SIMEX.estimates[,1]+1
b = mod.sim$SIMEX.estimates[,3]
plot(l[-1],b[-1],ylab="",xlab="",pch=19,ylim=range(b),xlim=range(l))
mtext(side=2,"Causal estimate",line=2.5,cex=1.5)
```

```
mtext(side=1,expression(1+lambda),line=2.5,cex=1.5)
points(c(1,1),rep(Fit2$coef[2],2),cex=2,col="blue",pch=19)
points(c(0,0),rep((mod.sim$coef[2]),2),cex=2,col="blue",pch=3)
legend("bottomleft",c("Naive MR-Egger","MR-Egger (SIMEX)"),
    pch = c(19,3),cex=1.5,bty="n",col=c("blue","blue"))
lsq = l^2; f   = lm(b~l+lsq)
lines(l,f$fitted)
```

References

[1] R. DerSimonian. and N. Laird. Meta-analysis in clinical trials. *Controlled Clinical Trials*, **7**:177-188, 1986.

[2] R.C. Paule and J. Mandel. Consensus values and weighting factors. *J Res Natl Bur Stand*, **87**:377-85, 1982.

[3] R. DerSimonian and R. Kacker. Random effect models for meta-analysis of clinical trials: an update. Contemporary Clinical Trials, **28**:105-114, 2007.

[4] J. Bowden, G. Davey Smith, and S. Burgess. Mendelian randomization with invalid instruments: effect estimation and bias detection through Egger regression. *International Journal of Epidemiology*, **44**:512-525, 2015.

[5] J.R. Cook and L.A. Stefanski. Simulation-extrapolation estimation in parametric measurement error models. *Journal of the American Statistical Association*, **89**:1314-1328, 1994.

[6] J.W. Hardin, H. Schmiediche, and R.J. Carroll. The simulation extrapolation method for fitting generalized linear models with additive measurement error. *Stata Journal*, **3**:373-385, 2003.

[7] W. Lederer and H. Kchenhoff. A short introduction to the SIMEX and MC-SIMEX. *R news*, **6**:26-31, 2006.

Additional Tables and Figures

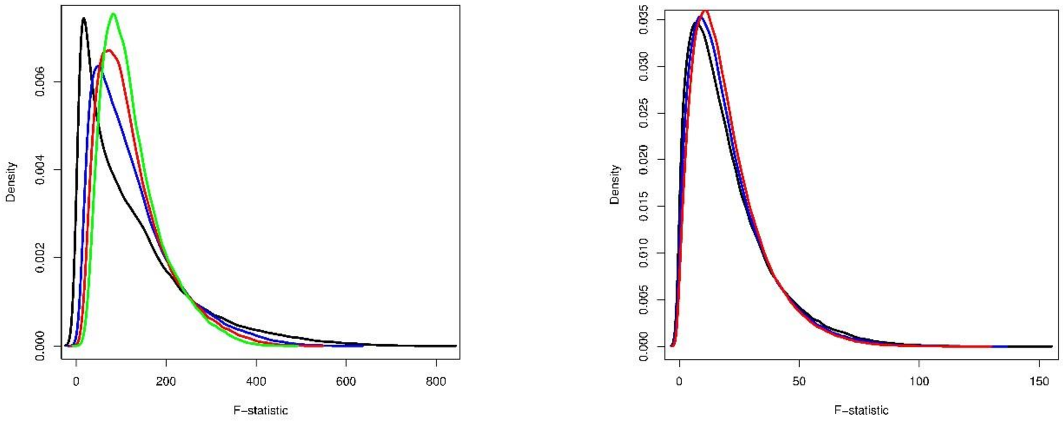

Figure 7: Left: Distribution of F-statistics for the simulated SNP-exposure data underlying the results in Table 1. $\overline{F}$ = 125 and I_{GX}^2 = 95% (black line), 90% (blue line), 85% (red line) and 75% (green line). Right: Distribution of F-statistics for the simulated SNP-exposure data underlying the results in Table 2. $\overline{F}$ = 20 and I_{GX}^2 = 60% (black line), 50% (blue line), 40% (red line).

	Uniform distribution bounds			
	σ_{Xj}		γ_j	
I_{GX}^2	lower	upper	lower	upper
95%	0.027	0.057	0.100	0.700
90%	0.027	0.052	0.200	0.600
85%	0.027	0.052	0.248	0.552
75%	0.026	0.051	0.291	0.509
60%	0.065	0.139	0.200	0.600
50%	0.064	0.138	0.235	0.565
40%	0.064	0.136	0.269	0.531

Table 4: Parameter ranges used to simulate SNP-exposure associations $\hat{\gamma}_1,\ldots,\hat{\gamma}_{25}$ for each I_{GX}^2 value given $\overline{F}$ = 125 (rows 1:4) and $\overline{F}$ = 20 (rows 5:7).
